# Mutations, Clinical Findings and Survival Estimates in South American Patients with X-Linked Adrenoleukodystrophy

**DOI:** 10.1371/journal.pone.0034195

**Published:** 2012-03-29

**Authors:** Fernanda dos Santos Pereira, Ursula Matte, Clarissa Troller Habekost, Raphael Machado de Castilhos, Antonette Souto El Husny, Charles Marques Lourenço, Angela M. Vianna-Morgante, Liane Giuliani, Marcial Francis Galera, Rachel Honjo, Chong Ae Kim, Juan Politei, Carmen Regla Vargas, Laura Bannach Jardim

**Affiliations:** 1 Gene Therapy Center, Hospital de Clínicas de Porto Alegre, Porto Alegre, Brazil; 2 Medical Genetics Service, Hospital de Clínicas de Porto Alegre, Porto Alegre, Brazil; 3 Post-Graduation Program in Medical Sciences, Universidade Federal do Rio Grande do Sul, Porto Alegre, Brazil; 4 Post-Graduation Program in Genetics and Molecular Biology, Universidade Federal do Rio Grande do Sul, Porto Alegre, Brazil; 5 Department of Analysis, Universidade Federal do Rio Grande do Sul, Porto Alegre, Brazil; 6 Department of Internal Medicine, Universidade Federal do Rio Grande do Sul, Porto Alegre, Brazil; 7 Hospital de Clinicas de Ribeirão Preto, Universidade de São Paulo, São Paulo, Brazil; 8 Department of Genetics and Evolutionary Biology, Institute of Biosciences, Universidade de São Paulo, São Paulo, Brazil; 9 Department of Genetics, Children Institute, Universidade de São Paulo, São Paulo, Brazil; 10 Department of Pediatrics, Universidade Federal do Mato Grosso do Sul, Campo Grande, Brazil; 11 School of Medicine, Universidade de Cuiabá, Cuiabá, Brazil; 12 Neuromuscular Disorders and Neuropathic Pain Section, Neurology Department, Hospital Juan A. Fernández, Buenos Aires, Argentina; Oslo University Hospital, Norway

## Abstract

In this study, we analyzed the *ABCD1* gene in X-linked adrenoleukodystrophy (X-ALD) patients and relatives from 38 unrelated families from South America, as well as phenotypic proportions, survival estimates, and the potential effect of geographical origin in clinical characteristics.

**Methods:**

X- ALD patients from Brazil, Argentina and Uruguay were invited to participate in molecular studies to determine their genetic status, characterize the mutations and improve the genetic counseling of their families. All samples were screened by SSCP analysis of PCR fragments, followed by automated DNA sequencing to establish the specific mutation in each family. Age at onset and at death, male phenotypes, genetic status of women, and the effect of family and of latitude of origin were also studied.

**Results:**

We identified thirty-six different mutations (twelve novel). This population had an important allelic heterogeneity, as only p.Arg518Gln was repeatedly found (three families). Four cases carried *de novo* mutations. Intra-familiar phenotype variability was observed in all families. Out of 87 affected males identified, 65% had the cerebral phenotype (CALD). The mean (95% CI) ages at onset and at death of the CALD were 10.9 (9.1–12.7) and 24.7 (19.8–29.6) years. No association was found between phenotypic manifestations and latitude of origin. One index-case was a girl with CALD who carried an *ABCD1* mutation, and had completely skewed X inactivation.

**Conclusions:**

This study extends the spectrum of mutations in X-ALD, confirms the high rates of *de novo* mutations and the absence of common mutations, and suggests a possible high frequency of cerebral forms in our population.

## Introduction

X-linked adrenoleukodystrophy (X-ALD, OMIM #300100) is a neurodegenerative disease characterized by great clinical expression variability even within the same family. Several phenotypes are recognized in males according to the age of onset, affected organs and rate of the progression of neurologic symptoms. The X-ALD clinical spectrum ranges from the rapidly progressive childhood cerebral form (CALD), which typically leads to severe disability and death during the first decade, to the milder adrenomyeloneuropathy (AMN) that usually manifests between ages 20 and 30 years and may be compatible with survival into the eighth decade, to pure Addison's disease [1]. Whereas AMN is characterized mainly by a noninflammatory “dying-back” axonopathy, involving the long spinal tracts, the inflammatory nature of the demyelinating lesion in CALD resembles those found in multiple sclerosis (MS), the most common central demyelinative disease. It is postulated that modifier genes or environmental factors are involved in the pathogenesis of these highly variable phenotypes [1], [2], [3].

X-ALD is caused by a defect in the gene for the adenosine triphosphate (ATP)-binding cassette protein, subfamily D, member 1 located on Xq28 (*ABCD1*) [1]. X-ALD protein (ALDP) [4], is a structural protein related to the transport of very long chain fatty acids (VLCFA) across peroxisome membranes. The *ABCD1* gene contains 10 exons, spanning 20 kb of genomic DNA and ALDP contains 745 amino acids.

Since its identification, 1236 mutations have been reported in the *ABCD1* gene of which 582 (47%) appear to be private (http://www.x-ald.nl). Mutations have been found throughout the entire gene, but they are not distributed evenly. There is a clustering of mutations in the transmembrane domain (47%), in the ATP-binding domain (34%), and in exon 5 (8%), which is not part of any of these domains. The remaining 11% of mutations are spread throughout other parts of the gene. No promoter mutations or complete gene deletions have been reported [5].

The main biochemical abnormality associated with X-ALD is the accumulation of unbranched saturated VLCFA in plasma and tissues, due to impaired β-oxidation in peroxisomes. The increase in VLCFA levels provides a reliable diagnostic tool for prenatal and postnatal identification of affected males. In females, however, VLCFA levels present false-negative results in around 20% of the obligate carriers [1]. Mutation analysis is therefore the best approach in order to improve genetic counseling to the families.

X-ALD is the most common peroxisomal disorder with a hemizygote frequency of 1∶21,000 in USA [6] and of at least 1∶35,000 in South Brazil [7]. Although no differences in distribution of mutations were found among different populations [5], [8], [9], [10], little is known about differences in X-ALD epidemiology in particular countries and continents. Specially, there is a relative lack of knowledge about the X-ALD epidemiology on subtropical regions of the world. This knowledge may not only help these populations, but may also help the identification of environmental factors that may modify X-ALD phenotype.

The present study aims to describe the *ABCD1* mutations in a case series of X-ALD families from South American patients, most of them Brazilian individuals, the rate of *de novo* mutations, phenotypes and survival estimates in the affected males found in this population.

## Materials and Methods

### Ethics Statement

The present work has been approved by the Ethics Committee from the institution at which the work was performed - Comissão de Ética em Pesquisa do Hospital de Clínicas de Porto Alegre -, which follows the Code of ethics of the World Medical Association (Declaration of Helsinki) and the standards established by the author's Institutional Review Board and granting agency. We have obtained written informed consent from all participants involved in the study.

### Patients

Patients originated from Brazil, Argentina and Uruguay, were previously diagnosed by VLCFA analysis. Most of them were followed-up in the main institution where the present study was carried out, while others were ascertained by their physicians in other sites. Families received genetic counseling and appropriate management, as described elsewhere [7]. These families were invited to participate in the present study, which was approved by the local Ethics Committee. Variables such as age, age at onset of symptoms, age at death, male phenotypes, genetic status (in women, such as obligate carriers), the family and the latitude of origin were also studied.

### Methods

After written consent, blood was collected from the index-case and DNA was extracted by the salting out procedure [11]. Using 10 PCR reactions it was possible to screen the entire coding sequence of the *ABCD1* gene and intron-exon boundaries using the protocol described by Boehm et al [12]. All samples were screened by single strand conformational polymorphism (SSCP) analysis followed by automated DNA sequencing to establish the specific mutation in each family. The different conditions used for SSCP were: 6% and 8% polyacrilamide-agarose gel eletrophoresis(PAGE) at room temperature, 8% and 10% PAGE at 4°C. These conditions were chosen at random. Amplicons with mobility shift were purified with Exo-SAP (GE Healthcare) and submitted to automated sequencing on ABI 3100 Genetic Analyzer using BigDye v3.0 (Life Technologies). Mutations were confirmed by reverse strand PCR sequencing. Pathogenicity of novel missense mutations was assessed by in silico analysis using PolyPhen (http://genetics.bwh.harvard.edu/pph/) and SIFT (http://sift.jcvi.org) based on aminoacid sequence NP_000024. Family relatives were screened for the specific mutation by PCR and automated sequencing.

The percent of mutations assigned at each exon was compared to those described in the literature at http://www.x-ald.nl. (17.10.2011). The mutations were also mapped according to the protein domains described by Kumar et al [13].

When an affected male was no longer available, an obligate carrier or a female relative with elevated VLCFA was chosen as the family's index case.

Patient characteristics are given as mean ± SD and range. Categorical variables such as normal, homozygote women versus heterozygote women were compared through chi-square test. Kaplan-Meyer curves were used to describe survival until disease onset, according to phenotypes, and to describe survival, in CALD. All tests were 2-sided. Test results were considered significant at the 0.05 level.

## Results

From 2007 to 2011, thirty-eight families entered the study. Of those, thirty-six were Brazilian families, 20 of them originating from Rio Grande do Sul, the Southern most state of Brazil. The clinical characteristics of some of these latter families have been previously reported [7]. We have also recruited one family from Uruguay and one family from Argentina to the present case series. Twenty-four families lived around parallel 30th South (Capricorn tropic), whereas 14 families lived between Equator and parallel 20th South.

### Molecular results

Sequencing of the exons with mobility shift detected by SSCP, revealed thirty-six different sequence variations in the 38 families (Table 1). Twelve index-cases (or 31.5% of the total sample of families) carried new mutations. The other 26 carried 24 mutations already described in the literature: 14 were missense mutations and 10 were nonsense or frameshift mutations. Insertion/deletion of one amino acid, or major deletions were not detected among our patients.

**Table 1:** Mutations found in the present study.

Family/Index case	Phenotype at diagnosis	Mutation	Exon/IVS	Mutation type	Effect on protein (cDNA)	Effect on protein (mRNA)	Protein localization	Origin of mutations	Origin of family
1/Female	asymptomatic	p.Gly512Ser (Feigenbaum V et al. 1996)	E6	Missense	c.1534G>A	GGC>AGC	NBF	***de novo***	Southern Brazil
2/Female	asymptomatic	p.Ser606Leu (Fanen P *et al.*, 1994)	E8	Missense	c.1817C>T	UCG>UUG	NBF	Inherited	Southern Brazil
3/Male	AMN	p.Trp601X (Gartner J et al.,1998)	E8	Stop codon	c.1802C>A	Truncated	NBF	Inherited	Southern Brazil
4/Female	asymptomatic	p.Arg617His (Fanen P *et al.*, 1994)	E8	Missense	c.1850G>A	CGC>CAC	NBF	ND	Southern Brazil
5/Male	AMN	**p.Pro623Leu^#^**	E9	Missense	c.1868C>T	CCC>CUC	NBF	Inherited	Southern Brazil
6/Male	AO	p.Trp326X (Barcelo A et al, 1996)	E2	Stop codon	c.978G>A	Truncated	TMD	Inherited	Southern Brazil
8/Female	asymptomatic	**p.Glu577X^#^**	E7	Stop codon	c.1729G>T	Truncated	NBF	Inherited	Southern Brazil
9/Male	asymptomatic	p.Arg554His (Smith KD et al., 1999)	E7	Missense	c.1661G>A	CGU>CAU	NBF	Inherited	Southern Brazil
10/Male	CALD	p.Arg518Gln (Imamura A et al., 1997)	E6	Missense	c.1553G>A	CGG>CAG	NBF	Inherited	Southern Brazil
11/Male	AO	**p.Tyr33_Pro34fsX34^#^**	E1A	Frameshift+stop codon	c.99_102delC	Truncated	-	Inherited	Southern Brazil
12/Female	asymptomatic	p.Gly266Arg (Fuchs S et al., 1994)	E7	Missense	c.1653insG	Truncated	TMD	ND	Southern Brazil
20/Male	CALD	**p.Arg538fs^#^**	E6	Frameshift	c.1614_1615dup27	Elonged	NBF	***de novo***	Southern Brazil
21/Male	CALD	**p.Ala232fsX64^#^**	E2	Frameshift+stop codon	c.696_697del11	Truncated	TMD	Inherited	Southern Brazil
22/Male	CALD	**p.Trp137fsX57^#^**	E1B	Frameshift+stop codon	c.411_412insC	Truncated	TMD	Inherited	Northern Brazil
23/Male	asymptomatic	p.Trp679X (Waterham HR et al, 1998)	E10	Stop codon	c.2037G>A	Truncated	NBF	ND	Southern Brazil
24/Male	AO	p.Tyr296Cys (Takano H et al., 1999)	E2	Missense	c.887A>G	UAU>UGU	TMD	Inherited	Southern Brazil
27/Male	CALD	**p.Leu628Glu^#^**	E9	Missense	c.1883T>A	CUG>GAG	NBF	Inherited	Southern Brazil
29/Male	CALD	p.Pro546fsX? (Fanen P et al. 1994)	IVS6	Frameshift+stop codon	IVS+1g>a	Splicing error ?	NBF	Inherited	Northern Brazil
31/Male	CALD	p.Arg518Gln (Imamura A et al., 1997)	E6	Missense	c.1553G>A	CGG>CAG	NBF	***de novo***	Southern Brazil
32/Male	CALD	p.Arg401Trp (Takano H et al., 1999)	E3	Missense	c.1201C>T	CGG>UGG	-	ND	Southern Brazil
33/Male	CALD	p.Thr632Pro (http://www.x-ald.nl)	E9	Missense	c.1894A>C	ACC>CCC	NBF	***de novo***	Southern Brazil
36/Male	CALD	p.Arg518Gln (Imamura A et al., 1997)	E6	Missense	c.1553G>A	CGG>CAG	NBF	Inherited	Northern Brazil
37/Male	CALD	p.Ser358X (Coll MJ et al., 2005)	E2	Stop codon	c.1073C>G	UCA>UGA	TMD	Inherited	Southern Brazil
38/Male	CALD	**p.Ile481Phe^#^**	E5	Missense	c.1441A>T	AUC>UUC	NBF	Inherited	Northern Brazil
39/Male	AMN	p.Arg389Gly (Krasemann EW et al., 1996)	E3	Missense	c.1165C>G	CGC>GGC	-	ND	Argentina
40/Male	AMN	p.Gln472fsX83 (Barceló A et al., 1994)	E5	Frameshift+stop codon	c.1415_1416delAG	Truncated	-	Inherited	Uruguay
41/Male	CALD	**p.Ala95fsX11^#^**	E1B	Frameshift+stop codon	c.283_284ins9	Elonged	TMD	Inherited	Southern Brazil
44/Male	CALD	p.Ser606Pro (Feigenbaum V et al. 1996)	E8	Missense	c.1816T>C	UCG>CCG	NBF	Inherited	Northern Brazil
45/Male	CALD	**p.Gln55X^#^**	E1A	Stop codon	c.163C>T	Truncated	-	Inherited	Northern Brazil
46/Male	CALD	p.Glu199Lys (http://www.x-ald.nl)	E1C	Missense	c.595G>A	GAG>AAG	TMD	ND	Northern Brazil
49/Male	CALD	p.Trp132X (Pan H et al., 2004)	E1B	Stop codon	c.396G>A	TGG>TGA	TMD	Inherited	Northern Brazil
50/Male	CALD	p.Glu477fsX80 (Valadares ER et al., 2011)	E5	Frameshift+stop codon	c.1430delA	Truncated	NBF	ND	Northern Brazil
51/Female	asymptomatic	p.Pro623fsX? (Kemp S et al., 1995)	IVS8	Frameshift+stop codon	c.1866-10G>A	Splicing error ?	NBF	ND	Northern Brazil
52/Male	CALD	**p.Arg401Gly^#^**	E3	Missense	c.1201C>G	CGG>GGG	-	Inherited	Southern Brazil
54/Female	CALD	**p.Ser358fsX42^#^**	E2	Frameshift+stop codon	c.1074_1075insA	Truncated	TMD	ND	Northern Brazil
55/Female	AMN	p.Gly510Ser (http://www.x-ald.nl)	E6	Missense	c.1528G>A	GGC>AGC	NBF	ND	Northern Brazil
56/Male	CALD	p.Asp200Asn (Takano H et al., 1999)	E1C	Missense	c.528G>A	GAC>AAC	TMD	Inherited	Northern Brazil
57/Male	CALD	p. Pro560Leu (Braun A et al., 1995)	E7	Missense	c.1679C>T	CCG>CTG	NBF	Inherited	Northern Brazil

The number of family: the registration number in records of our lab. AMN: adrenomyeloneuropaty; AO: Addison only; #: new mutations identified in this study; NBF: nucleotide-binding fold; TMD: Transmembrane Domin; ND: not determined. Southern Brazil: those families who lived near parallel 30^th^ South; Northern Brazil: those families who lived between Equator and parallel 20th South. Argentina and Uruguay lies on parallel 30^th^ or southern to it.

The already described p.Arg518Gln mutation [14] was found in three families from Rio Grande do Sul: one of these affected families was due to a *de novo* mutation in the maternal germ line. We can not rule out the possibility that the other two p.Arg518Gln pedigrees have a common ancestral origin, given their geographical proximity. All the other detected mutations were found in unique pedigrees.

Eight of the twelve new sequence variations were considered pathogenic mutations due to the creation of premature stop codon – either by nonsense mutation or as consequence of deletions, insertions or duplication (Table 1). The remaining four novel sequence variations (p.Pro623Leu, p.Leu628Glu, p.Ile481Phe and p.Arg401Gly), if not causative of the disease, were linked to X-ALD genotype by DNA study of hemizygous affected males. PolyPhen analysis of the four novel missense mutations considered three of them to be “probably damaging” p.Arg401Gly (PSIC score 3.071), p.Pro623Leu (PSIC score 3.379), and p.Leu628Glu (PSIC score 2.417), whereas p.Ile481Phe was considered “benign” (PSIC score 2.088). According to SIFT, all of these changes would not be tolerated.

**Figure 1:**
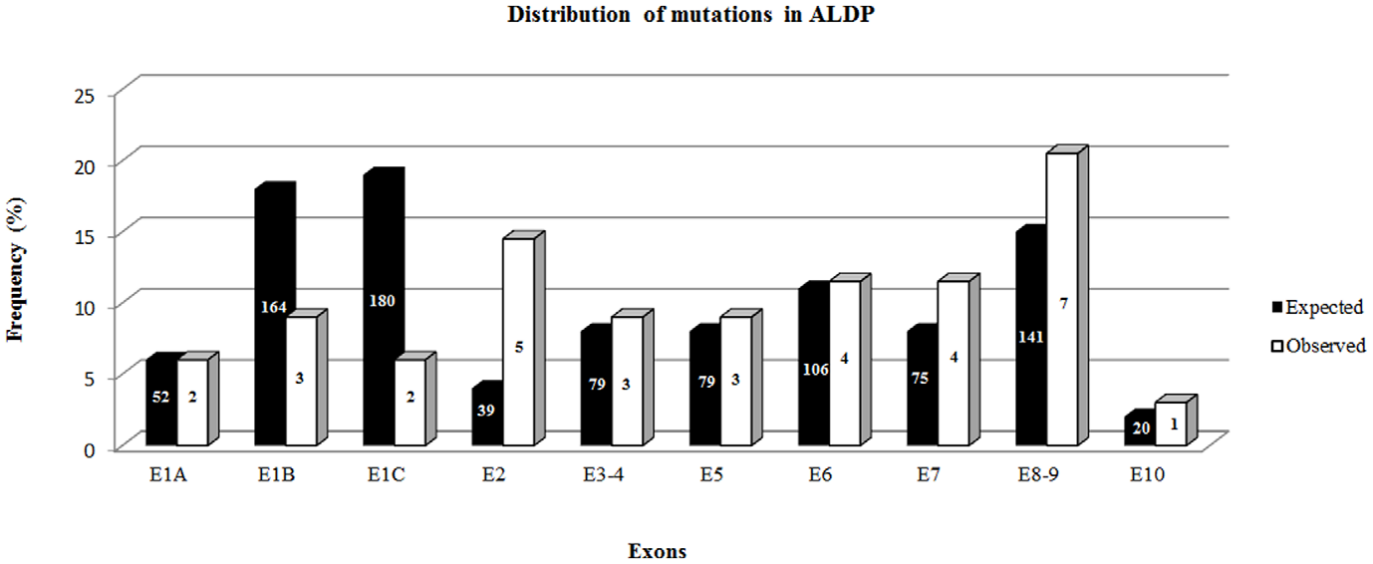
Distribution of the mutations observed in this series according to the amplicon regions (exons), and compared with the expected proportions ( http://www.x-ald.nl **).**

The incidence of mutations in exons 1A and in exon 3 to 6 were very similar to the reported in other populations (http://www.x-ald.nl). There seemed to be less mutations than expected in exons 1B and 1C, where we found 8.4% and 5.5% of our cases versus the expected 18% and 19%; and more mutations than expected in exon 2, with 14% of observations versus the expected 4%, (http://www.x-ald.nl) (Figure 1).

**Figure 2:**
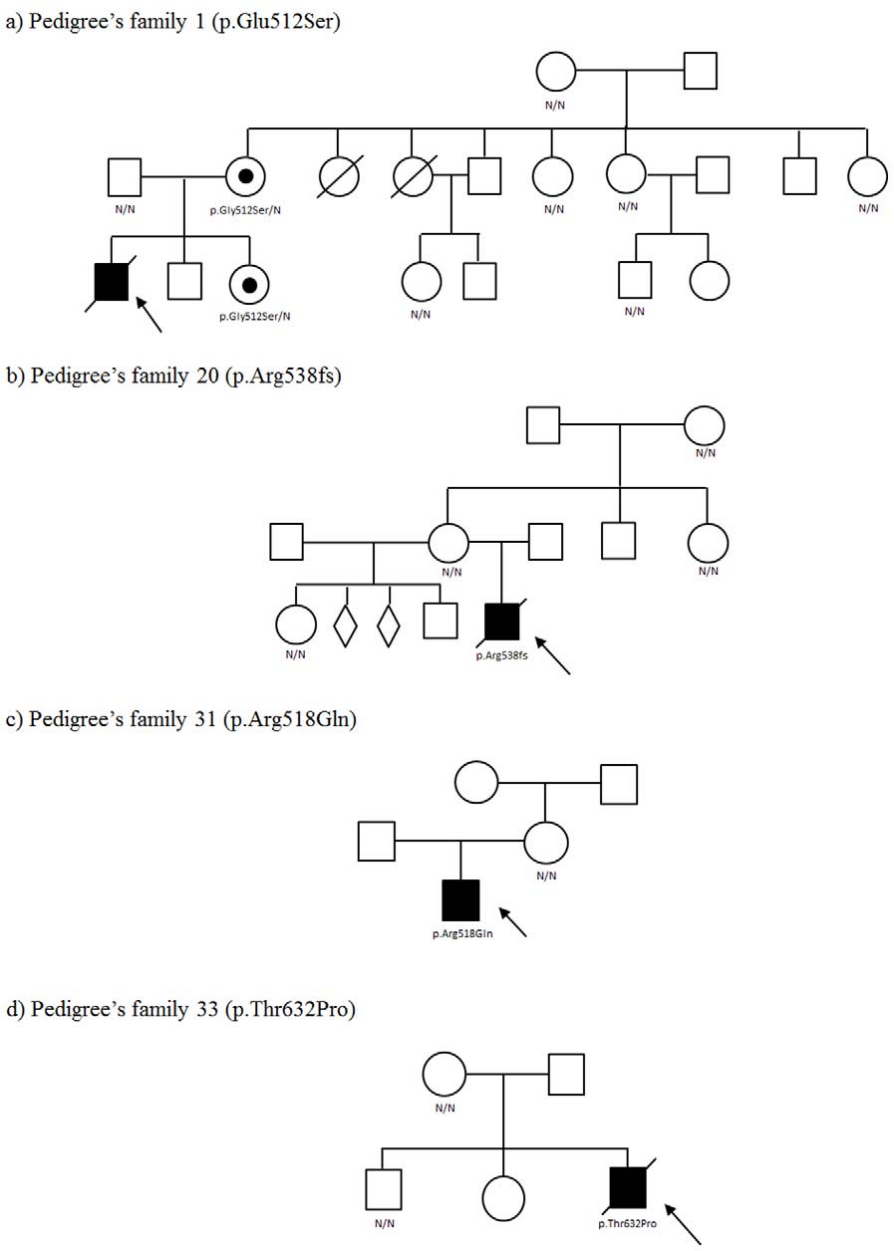
The four pedigrees where *de novo* mutations were detected.

In 35 families, information on the genetic status of the oldest transmitting mother was available. In this subset of families, we were able to define four *de novo* mutations (Figure 2).

From the remaining 34 families where the mutation was inherited, 21 men (2–53 years of age) and 77 women older than 18 years old were tested by molecular methods. Of those, thirty-eight females (or 49%) and thirteen males (or 62%) of the studied relatives also carried mutations. These proportions were in accordance with a priori mendelian risks (chi-square ns).

### Clinical characteristics of the present sample

Index-cases comprised 30 males and 8 females - six obligate carriers or biochemically proven heterozygotes, and two symptomatic women.

**Figure 3:**
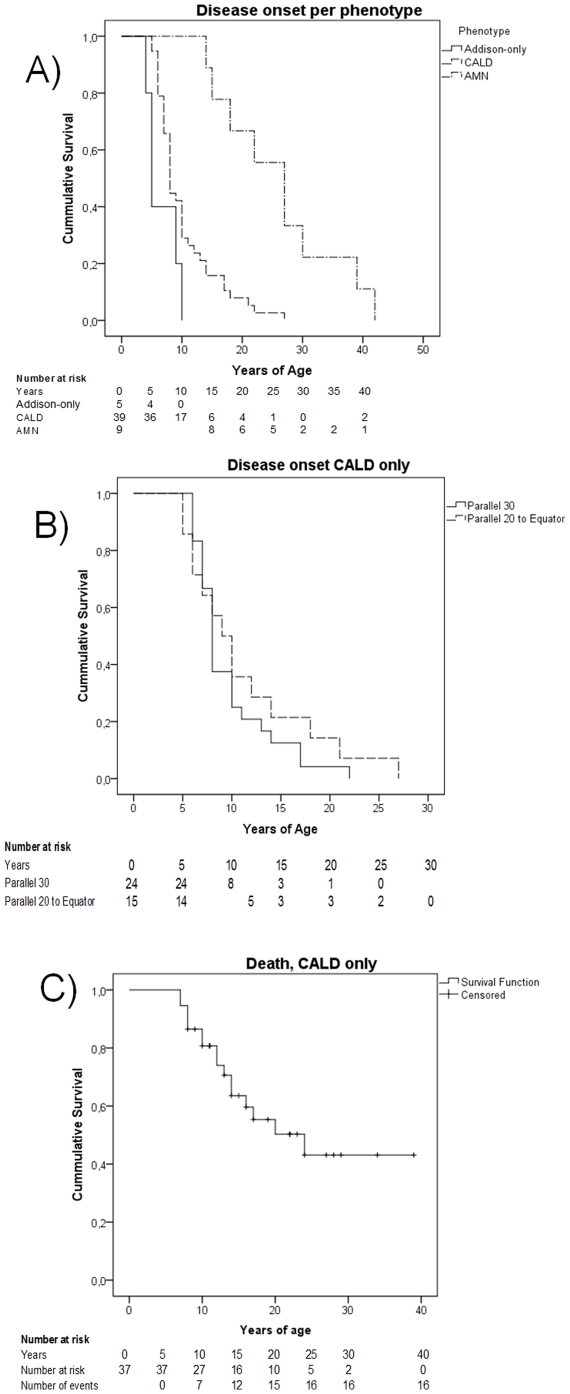
Kaplan–Meier survival curves. (A) Overall disease onset of the main phenotypes Addison-only, cerebral (CALD) and adrenomyeloneuropathy (AMN). Disease onset were significantly different (log rank test, p<0.001). (B) Disease onset of CALD, according to latitude of origin of the patient (ns). (C) CALD survival until death.

Clinical characteristics of the affected men, index-cases as well as the other affected relatives were described in Table 2. The family history of up to three generations, plus biochemical and molecular studies of the men alive identified 87 affected men in these 38 genealogies. At the end of this investigation, 6 asymptomatic, 6 Addison-only, 54 CALD (27 already deceased), and 14 AMN (3 already deceased) had been recognized. These phenotypes were distributed among the families, with no peculiar clustering in any pedigree (data no shown). Kaplan-Meyer curves on age at onset of each phenotype, and on survival of the cerebral forms were presented in Figure 3. No differences on proportion of phenotypes, on ages at onset or on survival (data not shown) of CALD were seen, according to the latitude of origin of the affected patient (Figure 3B and Table 1).

**Table 2:** Clinical characteristics of the affected men in the present families.

Clinical phenotypes	Number of cases (%)	Latitude of origin Parallel 30	Alive	Age at investigation (years)*	Age at onset (years)*	Age at death (years)*
Asymptomatic	6 (7%)		6/6	6.4*1.9–10.8*(2–14)	-	-
Addison-only	6 (7%)		5/6	15.2*5.3–25*(5–34)	7.4*5.4–9.4*(4–10)	10^a^
CALD	54 (62%)	39/54	22/49#	16.5*13.9–19*(7–39)	10.9*9.1–12.7*(5–27)	24.7^b^ *19.8–29.6*(7–24)
AMN	14 (16%)	11/14	11/14	40.2*32.5–47.8*(20–53)	26.4*20.3–32.5*(15–42)	*Unknown*
Unknown	7 (8%)		–			
Total	87 (100%)		44/75	19.7*16.2–32.2*(2–53)	12.5*10.2–14.7*(4–42)	-

CALD: Cerebral form, AMN: Adrenomyeloneuropathy.

* Mean, *CI 95%*, (range). Means estimated as survival functions (see Figure 3).

a one case,

b 16 cases.

# number of valid cases, 5 losses in follow up.

Two symptomatic women were the index-cases of their respective families. Case 55 was a 63 years old woman with a progressive, pure spastic paraplegia and urinary incontinence with 10 years of disease duration. There was no previous occurrence in the family. Her VLCFA profile was highly suggestive of a heterozygous state for X-ALD; the molecular analysis revealed the presence of the mutation p.GLy510Ser in one of her alleles (Table 2).

**Figure 4:**
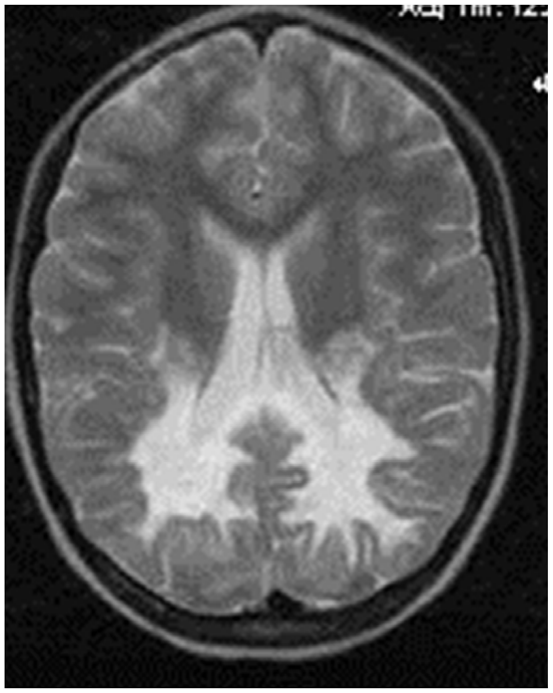
MRI of the female patient 54, showing the typical pattern of white matter abnormality found in X-ALD.

Case 54 was a 15 years old girl with clinical, biochemical and MRI abnormalities similar to those found in boys with the childhood cerebral phenotype (Figure 4). Motor and cognitive deterioration started at 6 years of age; VLCFA analysis was compatible with the heterozygous state (C26:0 of 2.02, normal range: 0.78 to 1.54; C26:0-C22:0 of 0.051, normal range: 0.01 to 0.03). She had completely skewed X-inactivation in blood cells, as determined by the human androgen receptor locus (HUMARA) methylation assay. Her G-banded karyotype was normal and the array-comparative genome hybridization (a-CGH) analysis revealed no X chromosome copy-number alterations (44K X-chromosome platform, design 2008, Agilent Technologies, Santa Clara, USA). Gene sequencing of this girl revealed the presence of the mutation p.Ser358fsX42 in one of her alleles (Table 1), probably on the active X chromosome.

### Genotype/Phenotype relationships

Some families were considered informative regarding the detected phenotypes: families with only CALD patients (more than one CALD patient), and heterogeneous families (with at least one CALD and one AMN). After that, the affected domain of the ALDP was defined: only NBF (20 families) and TMD (10 families) domains were considered. Distribution of these domains among the families characterized as CALD or heterogeneous was equal (ns, chi-square).

## Discussion

The present results probably describe the first case series of South American mutations related to X-ALD. Although it is usually said that all ethnic groups are affected by this disease, there is scarce knowledge about differences in frequency of X-ALD phenotypes or genotypes in our region, as well as in other subtropical regions of the world. Our results suggest some peculiarities such as a higher incidence of mutations in exons 2 and 8–9, and of cerebral forms, than the expected.

More than a thousand different mutations at *ABCD1* gene have been related to X-ALD. There are no common mutations for this disease: the most recurrent in other reports was p.Gln472fsX555, a frameshift mutation detected in 6.4% of cases recorded. At least 47% of all reported mutations have been found in single pedigrees in the world (http://www.x-ald.nl). Our rate of 34% of new mutations is in agreement with this overall picture, moreover if we remember that even the only recurrent mutation in our case series (p.Arg518Gln) has emerged at least once from a *de novo* phenomenon (Table 1 and Figure 2).

We have found 4/35 *de novo* mutations, or 10% (Figure 2). In a previous large series, the rate of the detected *de novo* mutations had been of 5% in affected male probands [7]. The actual numbers might be much higher, if at least three generations (up to the grand-mothers) would be checked. The absence of any frequent mutation, even in restricted populations, states against the existence of distant common ancestors for contemporary patients. The same phenomenon appeared in our sample.

In our sample, the proportions of missense, frameshift and nonsense mutations were similar to those found worldwide (http://www.x-ald.nl). In contrast, a possible cluster of mutations at two amplicon regions, exons 2 and 8–9, was observed (Figure 1).

The occurrence of CALD in a girl with a skewed X-inactivation in our case series deserves consideration. The present female patient resembled the one reported by Hershkovitz et al [15]. Both girls have clinical, biochemical and MRI abnormalities similar to those seen in boys with CALD. Two important differences must be stressed, however. Our patient did not have a positive family history of X-ALD, nor an X chromosome deletion.

Skewing of inactivation is frequently observed in heterozygotes for *ABCD1* mutations, and it was found to correlate with neurological manifestations [16]. As far as we know, skewing *per se* was never related to CALD in a woman. The occurrence of CALD in our patient points to the X chromosome bearing the c.1074–1075insA mutation in *ABCD1* to be the active one, rendering “the patient totally deficient in ALDP and equivalent to an affected male” [15].

Our phenotype proportions in males seemed to differ from the expected ones, reported in literature. In the present study, all efforts to obtain a complete family history of up to three generations were done, plus biochemical and molecular studies of the men alive. We identified 87 affected men in 38 genealogies (including the *de novo* pedigrees), or 2.2 cases per family, a rate similar to those found by others in larger series [6]. However, our rates of CALD (62%) were higher than the expected 45–57% [1], [17], [18]. We are aware that underdiagnosis would be the best explanation to the present numbers, moreover because only 6% of our series comprised Addison-only disease (expected to be between 8 and 20%). However, if underdiagnosis of AMN and Addison-only was operating here, we would be unable to explain why the molecular results obtained in male relatives studied after genetic counseling of the nuclear family were near as those expected *a priori*. If the clinical characterization of our sample was insufficient, a higher proportion of carriers found by chance would be expected.

If the present observation of a high proportion of CALD in our sample was correct, the hypothesis of an environmental factor should be considered. Head trauma has been postulated as a risk factor for the cerebral form in X-ALD [17], but we did not observe such phenomena. Inflammation and altered immune responses are present in CALD, whose demyelinating lesion is very similar to those seen in multiple sclerosis (MS) [19], [20]. Among other factors, latitude is clearly related to MS prevalence, and environmental modifiers which vary with latitude, such as ultraviolet radiation, have been postulated to MS [21]. By analogy, we speculated if differences in latitude could also be related to a higher prevalence and/or natural history of CALD. Our data did not support this association, though the small latitude range of the present observation might have precluded a final conclusion.

We believe that further studies on X-ALD prevalence, mutation patterns, natural history and clinical course after bone marrow transplantation in countries outside Europe and USA are necessary. This knowledge will further help the management of these families, as well as may help the understanding of disease mechanisms.
